# A Novel Approach for Fully Automated, Personalized Health Coaching for Adults with Prediabetes: Pilot Clinical Trial

**DOI:** 10.2196/jmir.9723

**Published:** 2018-02-27

**Authors:** Estelle Everett, Brian Kane, Ashley Yoo, Adrian Dobs, Nestoras Mathioudakis

**Affiliations:** ^1^ Division of Endocrinology, Diabetes and Metabolism Department of Medicine Johns Hopkins University Baltimore, MD United States; ^2^ Reading Health Physician Network Reading, PA United States

**Keywords:** mobile health, prediabetes, HbA _1c_, weight loss, blood glucose

## Abstract

**Background:**

Prediabetes is a high-risk state for the future development of type 2 diabetes, which may be prevented through physical activity (PA), adherence to a healthy diet, and weight loss. Mobile health (mHealth) technology is a practical and cost-effective method of delivering diabetes prevention programs in a real-world setting. Sweetch (Sweetch Health, Ltd) is a fully automated, personalized mHealth platform designed to promote adherence to PA and weight reduction in people with prediabetes.

**Objective:**

The objective of this pilot study was to calibrate the Sweetch app and determine the feasibility, acceptability, safety, and effectiveness of the Sweetch app in combination with a digital body weight scale (DBWS) in adults with prediabetes.

**Methods:**

This was a 3-month prospective, single-arm, observational study of adults with a diagnosis of prediabetes and body mass index (BMI) between 24 kg/m^2^ and 40 kg/m^2^. Feasibility was assessed by study retention. Acceptability of the mobile platform and DBWS were evaluated using validated questionnaires. Effectiveness measures included change in PA, weight, BMI, glycated hemoglobin (HbA_1c_), and fasting blood glucose from baseline to 3-month visit. The significance of changes in outcome measures was evaluated using paired *t* test or Wilcoxon matched pairs test.

**Results:**

The study retention rate was 47 out of 55 (86%) participants. There was a high degree of acceptability of the Sweetch app, with a median (interquartile range [IQR]) score of 78% (73%-80%) out of 100% on the validated System Usability Scale. Satisfaction regarding the DBWS was also high, with median (IQR) score of 93% (83%-100%). PA increased by 2.8 metabolic equivalent of task (MET)–hours per week (SD 6.8; *P*=.02), with mean weight loss of 1.6 kg (SD 2.5; *P*<.001) from baseline. The median change in A_1c_ was −0.1% (IQR −0.2% to 0.1%; *P*=.04), with no significant change in fasting blood glucose (−1 mg/dL; *P*=.59). There were no adverse events reported.

**Conclusions:**

The Sweetch mobile intervention program is a safe and effective method of increasing PA and reducing weight and HbA_1c_ in adults with prediabetes. If sustained over a longer period, this intervention would be expected to reduce diabetes risk in this population.

**Trial Registration:**

ClincialTrials.gov NCT02896010; https://clinicaltrials.gov/ct2/show/NCT02896010 (Archived by WebCite at http://www.webcitation.org/6xJYxrgse)

## Introduction

### Scope of the Problem

Prediabetes, a high-risk state for future type 2 diabetes, is a global epidemic with increasing prevalence in both developing and developed countries [1]. Among adults, the prevalence of prediabetes in the United States, the United Kingdom, China, and India is 33.9%, 35.3%, 35.7%, and 10.3%, respectively [2-5]. Considering that the complications associated with type 2 diabetes begin at the prediabetes stage [6] and that more than half of individuals with prediabetes will eventually develop diabetes [7], efforts are urgently needed to intervene on this high-risk population. Regular physical activity (PA; 150 min per week), weight loss, and healthy diet are highly effective strategies for preventing or delaying the onset of type 2 diabetes [8-10]; however, randomized controlled trials (RCTs) of these interventions have required intensive one-on-one or group lifestyle coaching, which raises questions about the feasibility and scalability of implementing these interventions outside of research settings. For example, the landmark Diabetes Prevention Program (DPP), which consisted of a 16-session individual curriculum over 6 months in addition to supervised group exercise sessions, had estimated direct costs of approximately US $1400 per person annually (of which more than half of the cost was for staffing) [10,11].

Mobile health (mHealth) technologies potentially represent an ideal method to deliver diabetes prevention interventions on a large scale given the ability to reach sizable numbers of patients at substantially lower costs than human-based interventions. Various digitally supported interventions have been used for diabetes prevention and weight loss, including email, voice calls, SMS text messaging (short message service, SMS), Web-based applications, mobile apps, activity monitors, and telemedicine [12]. In fact, the Centers for Disease Control and Prevention (CDC) has recently started to recognize virtual programs as valid DPPs [13]. Most of these programs, which have shown promise in diabetes prevention, have required either in-person training [14] or have a major component of human coaching [15,16], which limits scalability and long-term adherence, as interventions that rely on human coaching require substantial human resources and professional training, as well as sustained time commitment on the part of participants.

### Study Intervention

Sweetch is a fully automated, personalized, artificial intelligence-based mHealth platform (Sweetch Health, Ltd) designed to promote adherence to PA, weight reduction, and diet guidelines for people with prediabetes. To sustain healthy life habits, Sweetch uses artificial intelligence (machine learning) to automatically translate various raw mobile phone data streams into insights about the user’s life habits. Completely free of human involvement, Sweetch presents personalized, contextual, just-in-time, just-in-place recommendations with the goal of guiding the user toward achieving his or her desired activity and weight reduction goals.

The Sweetch mobile platform is designed to initially address adherence to the PA component of diabetes prevention strategies, and as participants comply with the PA goal, a weight reduction goal is subsequently introduced. Evidence suggests that PA alone, independent of weight loss or adherence to dietary recommendations, is an effective diabetes prevention strategy. Large RCTs have shown that diabetes risk is (1) reduced substantially for participants who meet exercise goals even if failing to meet weight loss or diet goals [17], (2) is comparable in participants who meet exercise goals and those who meet both diet and exercise goals [8], and (3) that leisure time PA (LTPA) is associated with resolution of the metabolic syndrome [18]. A large systematic review of 300,000 participants revealed a 30% lower relative risk of type 2 diabetes in those with regular PA (defined as walking for 150 min per week at a brisk pace) compared with almost no walking, an association that persisted even after adjustment for body mass index (BMI) [19].

### Study Objectives

The objective of this pilot study was to determine the feasibility, acceptability, safety, and effectiveness of the Sweetch app in combination with a digital body weight scale (DBWS) in adults with prediabetes. As the Sweetch app is fully automated, a major objective of the pilot study was to calibrate the system (ie, explore which recommendation types work best and in what context). We hypothesized that the Sweetch intervention would result in clinically meaningful improvements in LTPA and body weight and potentially improvements in glycemic control over the follow-up period.

## Methods

### Study Design

This was a 3-month single arm, prospective, observational study conducted at two clinical sites within the Johns Hopkins Clinical Research Network: Johns Hopkins Hospital, a tertiary care academic medical center in Baltimore, Maryland, and a community internal medicine practice within the Reading Health System in Reading, Pennsylvania conducted from October 10, 2016 to November 17, 2017. The study was approved by the institutional review boards (IRBs) of both institutions, and written informed consent was obtained from all participants. The clinical trial was registered on ClinicalTrials.gov (NCT02896010).

Initially, the study was designed as an RCT with two arms, in which participants were assigned to receive either the Sweetch app alone or the Sweetch app in combination with a DBWS. Early on in the trial, it became clear that inclusion of the DBWS was necessary for the purpose of app calibration, as this allowed real-time correlation of weight data with PA measures; therefore, the design was revised to a single-arm observational study in which all subsequently enrolled participants were assigned to receive the Sweetch app and a DBWS. At the time of IRB approval of this change in the study design (February 7, 2017), 14 participants had received the app alone and were continued in the study according to their initial treatment assignment.

**Eligibility criteria for participants in the study.**

Eligibility criteria

- Carried a diagnosis of prediabetes (at least one of the following diagnostic criteria: impaired fasting glucose confirmed by a fasting glucose 100-125 mg/dL, impaired glucose tolerance confirmed by a 2-hour glucose of 140-199 mg/dL following a 75-gram oral glucose tolerance test, or glycated hemoglobin [HbA_1c_] 5.7% to 6.4%)
- Had a body mass index (BMI) between 24 to 40 kg/m^2^ or 22 to 40 kg/m^2^ for Asian individuals
- Were English speaking
- Had a mobile phone (Android or Apple 5S and above)

**Exclusion criteria for participants in the study.**

Exclusion criteria

- Any medical condition that prevented adoption of moderate intensity physical activity (defined as inability to walk at a 15-20 minute mile pace)
- Body weight >400 lb (as this weight exceeded the upper limit of the digital body weight scale)
- Any diagnostic criterion for diabetes mellitus
- Use of any glucose-lowering medication or weight-loss medication within the previous 3 months
- Current use of systemic glucocorticoids
- Use of antipsychotic medications
- Elevated liver enzymes (3 times upper limit of normal)
- Conditions that can result in spurious A_1c_ readings (eg, anemia or hemoglobinopathy)
- Severe mental illness or learning disability
- Current participation in another clinical trial

### Participants

Participants were recruited via various methods including clinician referral, chart review, on-site advertisements, flyers or posters, and social media advertisements. Textboxes 1 and 2 show the inclusion and exclusion criteria for participants in the study.

These exclusion criteria were selected because of the potential confounding effect they could have on glycemic control during the study. Participants who met the above criteria were subsequently screened by laboratory testing (if lab results were not available within 14 days before screening) and vital signs to confirm eligibility. An enrollment target of 50 participants was selected based on convention, as enrollment numbers of 50 to 100 participants are typical in pilot studies of exercise or weight loss interventions.

### Study Procedures

Participants who met the eligibility criteria were invited to return for a baseline visit within 14 days, at which time the Sweetch app was downloaded on the participant’s mobile phone, and the participant was registered within the app. If the participant was assigned to receive the DBWS, it was provided and synchronized to the Sweetch app.

Following this baseline visit, the participant returned to the clinic in 90 days (window of 76-120 days) for the final visit, at which time repeat laboratory tests (A_1c_ and fasting glucose) and vital signs were collected, and usability or satisfaction questionnaires were administered. Participants who developed any of the exclusion criteria after enrollment, logged out of the app, or removed the app from their mobile phone for a period of at least 14 consecutive days were dropped from the study, as data could not be collected from those participants. As it was not known a priori whether this intervention would be effective for reducing risk in adults with diabetes, from an ethical standpoint, all participants were referred to a local registered dietician (RD), who was also a certified diabetes educator (CDE), to receive lifestyle counseling at the baseline visit. In addition, a 1-page brochure related to diabetes prevention was provided.

Demographic information, past medical history, and medications were collected from participants at the baseline visit. A 5-item mobile phone usage and attitudes survey, adapted from the mobile phone domain of the Media and Technology Usage and Attitudes Scale [20], was administered at baseline (Multimedia Appendix 1). To assess the participant’s willingness to increase PA, a 4-item Physical Activity States of Change Questionnaire [21] was administered electronically upon participant’s registration within the app. Responses to this questionnaire are scored as previously described to classify the participant’s baseline PA stage of change category (precontemplation, contemplation, preparation, decision or action, and maintenance).

Biometric measures including blood pressure, height, weight, and waist circumference were measured at the baseline and final visits. Although most participants received a DBWS, only clinic-based weight measurements were used as outcome measures. Waist circumference was measured using a flexible measuring tape according to the technique recommended by the American Society for Nutrition [22]. Blood pressure was measured in accordance with recommendations by the American Heart Association [23]. A_1c_ and glucose measurements were obtained via a central laboratory serum specimen. Fasting glucose measurements were obtained after participant reported a minimum of 8 hours fast.

### Description of Sweetch Mobile Platform

Sweetch is a personal digital intervention program that seeks to help individuals lose weight and become more active with the goal of reducing their long-term risk of diabetes and other conditions associated with the metabolic syndrome. Sweetch’s core philosophy is that each individual has his or her own life habits, motivations, and pace of behavioral-change progress; therefore, generic recommendations to walk 10,000 steps or for 30 min a day and eat less carbohydrates may not produce sustainable and meaningful behavioral change for all individuals, especially in the long run. Sweetch uses machine learning to automatically translate raw data streams originating from the patient’s mobile phone and DBWS into insights about the individual’s life habit—schedule, activity patterns, driving and walking routes, surroundings, and more. Then, using advanced algorithms, Sweetch presents the user with personalized, contextual, just-in-time, just-in-place, recommendations that guide him or her toward achieving recommended activity, weight reduction, and diet goals in a way that fits the user’s real-world life habits. Sweetch’s technology learns what types of message result in better compliance for the specific user at a specific context (ie, day of week, time, location, effect of consecutive messages of different types, etc).

The Sweetch app translates behavioral change theory [24] into practice by the following means:

- breaking down the target of 150 min of PA per week into small segments throughout the day, making the goals more achievable. These small segments are personalized to each user on an on-going basis.
- providing direct feedback and encouragement when goals are met.
- shaping behavior by identifying and immediately reinforcing the target behavior.
- pointing out opportunities in the user’s daily routine for increased LTPA. Notifications constitute “teachable moments” that could result in sustained behavioral change.

**Figure 1:**
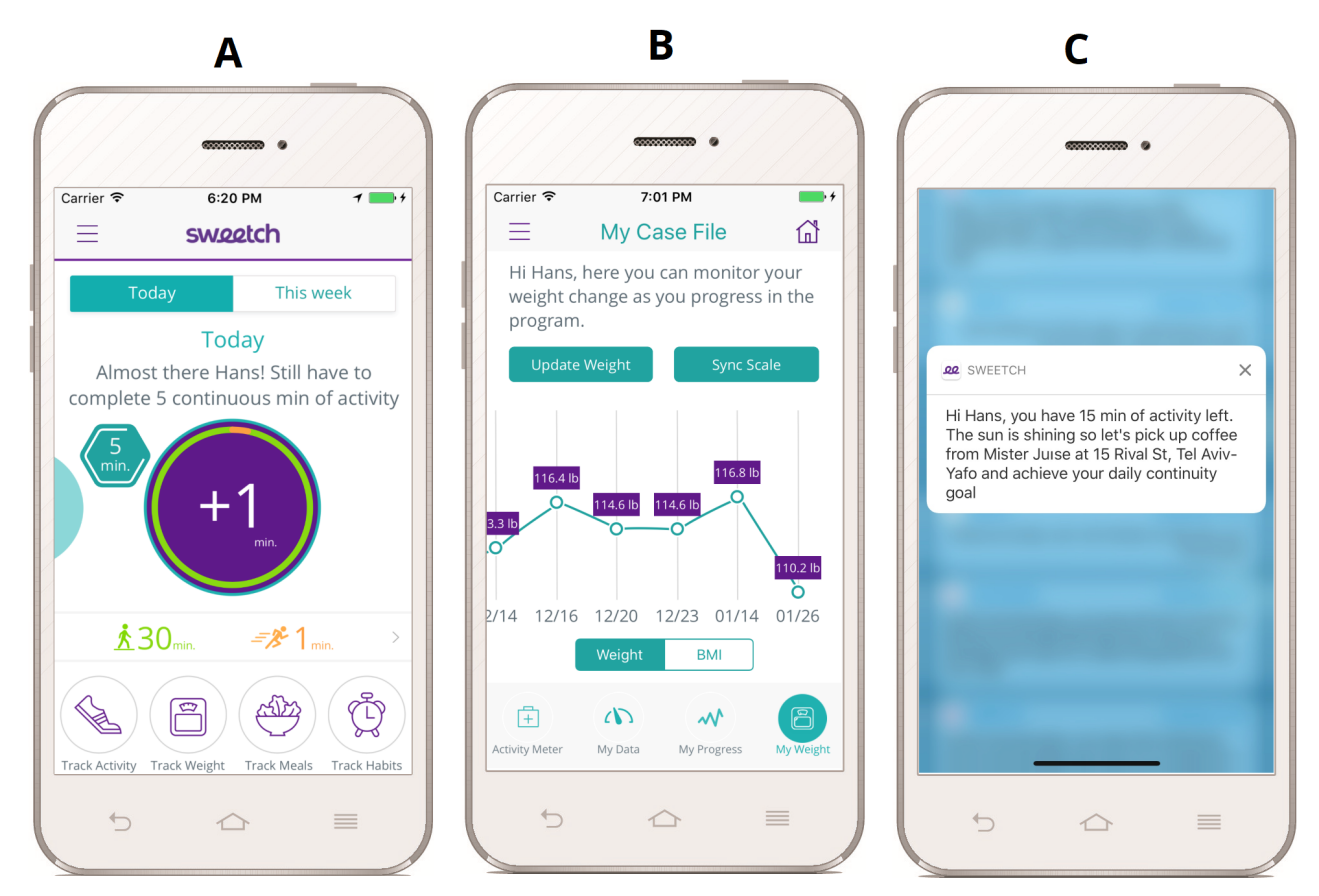
Features of Sweetch app. A. Activity Tracking on daily and weekly view, presenting remaining activity to be completed. B. Weight changes summarized graphically. C. Personalized push notification adapted based on actual life habits.

This approach aims for gradual progress, high level of personalization, and long-term adherence. The Sweetch app enables the participant to track activity progress on a daily and weekly view in the app, presenting remaining activity that needs to be completed (Figure 1). Weight change and goals are summarized graphically over time. Personalized push notifications are adapted based on actual life habits and sent to users, providing an actionable recommendation.

**Figure 2:**
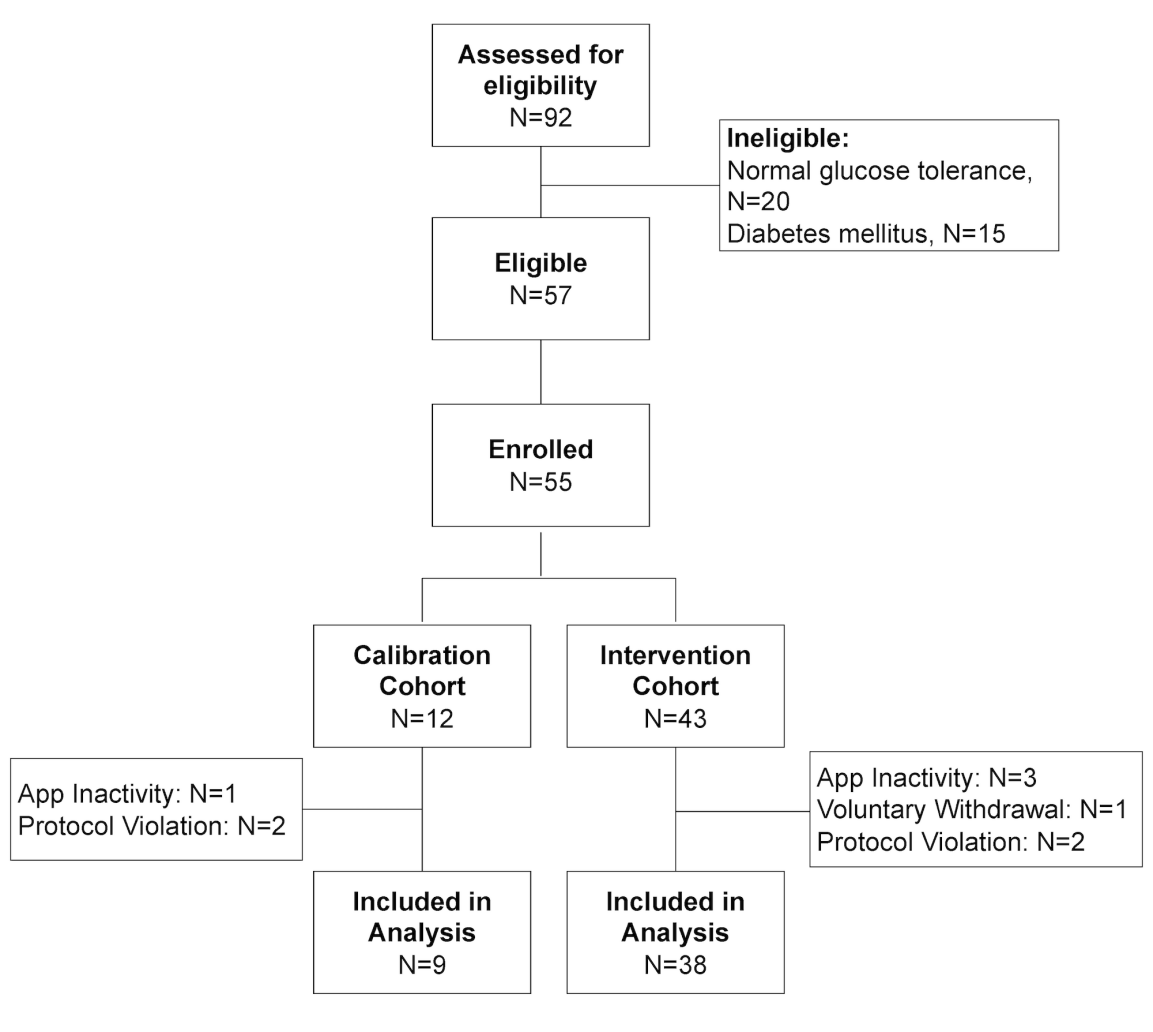
Study enrollment flowchart.

The novelty of the Sweetch mobile platform is that it optimizes, in real time and using fully automated algorithms, the messages each user gets so to achieve best possible compliance. Similarly, the personalized goals are continuously adapted based on the user's real-world behavior to best fit the user’s real-life capabilities. The Sweetch app uses a passive assessment approach to provide tailored recommendations with minimal or no engagement on the part of the user. A few examples of Sweetch’s tailored recommendations include (1) notifying the user to do activity only when the user’s calendar indicates available time, (2) recommending specific activity based on the user’s surrounding locations (park, coffee shops, etc) as reflected by the user’s real-time location, (3) sending weight notifications only when the user is at home, and (4) varying notification elements (positive, social, etc) in different sequences based on the user’s real-world reaction to different timing and types of messages.

### Digital Body Weight Scale

The Sweetch app communicates with a commercially available Bluetooth-enabled digital scale with a body composition analyzer (Shenzhen Unique, Ltd; Model No. CF369). The DBWS syncs weight data to the Sweetch app using Bluetooth technology (BLE 4.0) and supports communication from Android and iPhone operating system (iOS, Apple Inc) mobile phones.

### Calibration and Intervention Cohorts

This pilot study consisted of two cohorts: a calibration cohort and an intervention cohort (Figure 2). The earliest participants enrolled in the study were entered in a calibration cohort. Raw mobile phone data and weight measurements obtained from the DBWS were correlated with the clinic-based outcomes from the initial participants (weight change and change in glycemic measures) to continually adjust metrics of relevant PA that corresponded to the greatest improvements in body weight and glycemic measures. In addition, several refinements were made in the app during the calibration period, including the incorporation of the participant’s calendar data (indicating available time for PA), enhancing the sensitivity of the PA tracking, taking into consideration varying definitions of continuous activity as indicators of effective activity, and increasing app availability during different operating system modes (eg, suspended and terminated). An enhanced version was developed based on outcomes and usability or satisfaction responses of participants in the calibration cohort. In the analysis, any participant who received this enhanced version for 50% or more of their time in the trial was considered part of the intervention cohort.

### Outcome Measures

This pilot study evaluated measures of feasibility, acceptability, effectiveness, and safety. Feasibility was assessed by retention, defined as the proportion of enrolled participants who completed and adhered to the study protocol. Acceptability was evaluated at the final visit using a 10-item questionnaire with Likert scale responses regarding the Sweetch app, a six-item questionnaire regarding the DBWS, and a two-item questionnaire comparing the perceived benefit of the app with the DBWS on attainment of weight loss and PA goals. The app questionnaire was adapted from the validated System Usability Scale (SUS), a quick and reliable tool to evaluate a wide variety of technological products, including mobile devices [25]. The responses to the app questionnaire were rescored from 0 to 100, and a median score above 68 was deemed a priori to indicate above average user acceptability. The six-item questionnaire for the DBWS was developed specifically for this study. Responses were rescored from 30 to 100, and a median score of 68 and above was considered acceptable. The two-item questionnaire had four categorical responses comparing the app with DBWS on attainment of weight loss and activity goals. Satisfaction scores are reported for all participants who completed the study per protocol.

Effectiveness measures included changes in PA, weight, BMI, A_1c_, fasting glucose, and blood pressure. PA was evaluated using metabolic equivalent of task (MET)–hours, which was the same metric used in the DPP. MET-hours were calculated as the product of the duration and type of PA, weighted by an estimate of the metabolic equivalent of that activity, and summed per week, with the result expressed as the average MET-hours per week. Unlike the DPP, which used self-reported data on PA, calculations of MET-hours were based entirely on data captured automatically by the participant’s mobile phone (details provided in Multimedia Appendix 2). Effectiveness measures are reported for the intervention cohort only.

As the Sweetch app aims to increase LTPA and reduce weight gradually, the intervention was not expected to pose any serious risk of adverse events (AEs) for participants. Participants were instructed to call the research coordinator by phone or email in the event of an AE, in which case the principle investigator would contact the participant for details and adjudicate whether the event was related or unrelated to the intervention. At the final visit, participants were asked about any new medical problems or hospitalizations experienced during the study, whether they believed these to be related to the intervention, and the reasons why.

### Statistical Analysis

As this was a pilot study, formal sample size calculations were not performed. Participant data were statistically analyzed using Stata statistical software: release 13 (StataCorp LP). Summary statistics describing the baseline demographic characteristics, PA stage of change, and mobile phone usage attitudes score are provided in Table 1, with results reported for all enrolled participants and separately for the calibration and intervention cohorts. Normality of continuous variables was assessed using the Shapiro Wilk test. For normally distributed and nonnormally distributed continuous variables, respectively, mean (standard deviations) and median with interquartile range (IQR) are reported. Counts and frequencies are reported for categorical variables.

**Figure 3:**
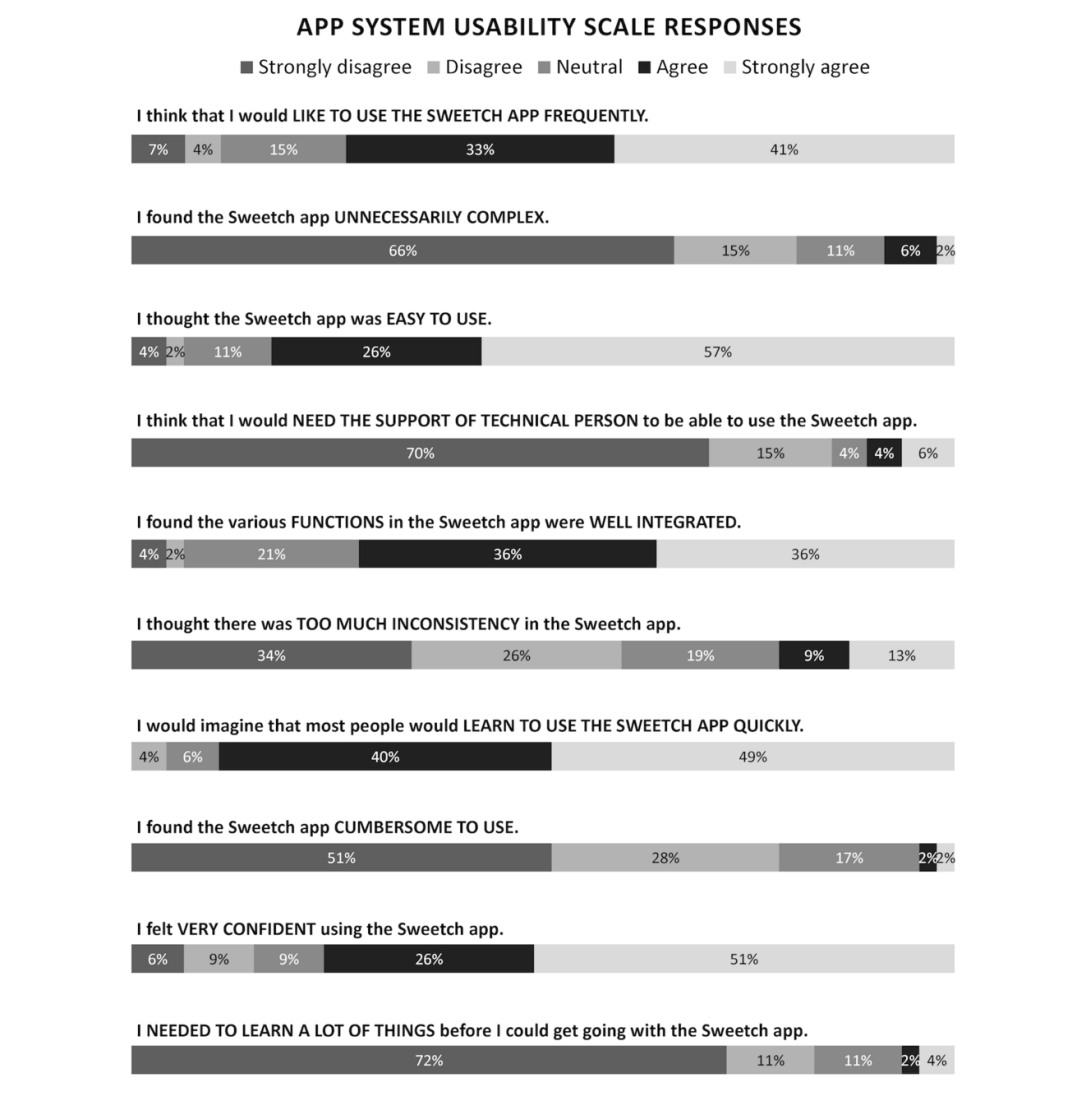
Sweetch app usability and satisfaction results of participants who completed study per protocol (in both calibration and intervention cohorts, N=47).

**Figure 4:**
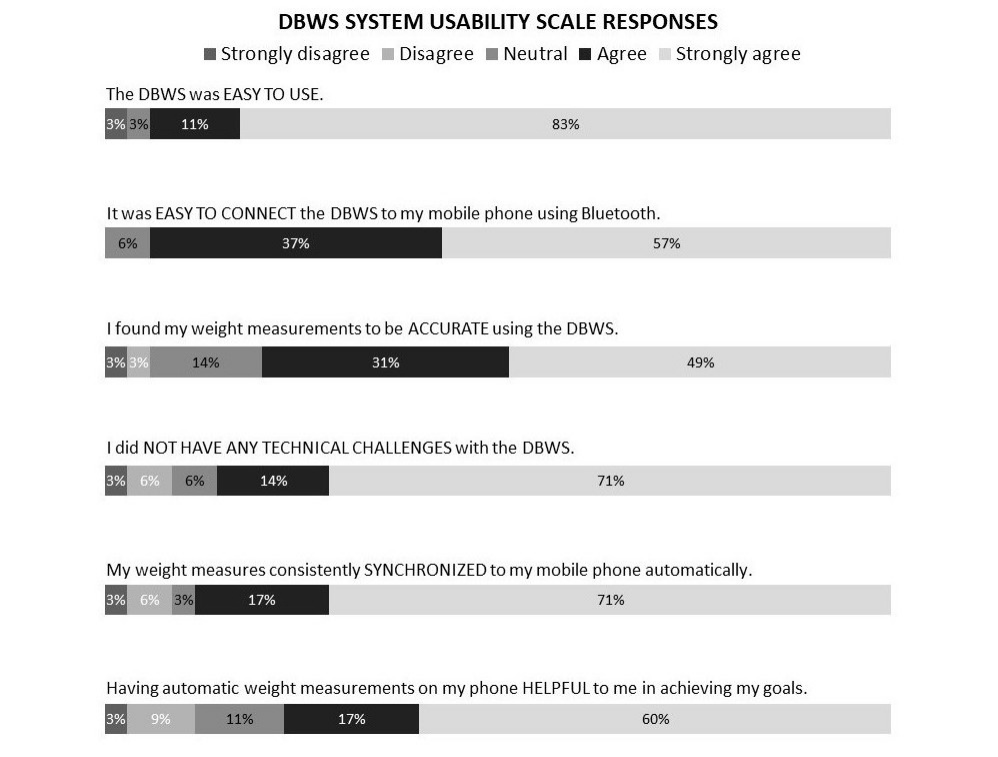
Digital body weight scale (DBWS) usability and satisfaction results of participants who completed study per protocol (in both calibration and intervention cohorts, N=35).

To estimate the statistical differences between the pre- and postintervention measurements in the intervention cohort, paired *t* test and Wilcoxon matched pairs (signed-rank) test were used for normally distributed and nonnormally distributed continuous variables, respectively (Table 2). A two-tailed *P* value <.05 was considered statistically significant. Change in PA (MET-hours per week) was calculated by subtracting the participant’s mean MET-hours per week of the last 2 full weeks on study from the mean MET-hours per week of the first full 2 weeks on study (weeks 2 and 3). For all other continuous outcome measures, the change from baseline was calculated by subtracting the value of the baseline visit from the final visit. For normally distributed variables (PA, weight, BMI, and waist circumference), the mean and SDs are reported for the baseline visit and final visit, and statistical significance in the mean of the paired differences (change from baseline) was evaluated using the paired *t* test. For nonnormally distributed variables (A_1c_, fasting glucose, systolic blood pressure, and diastolic blood pressure), the median and IQR are reported for the baseline and final visits, and statistical significance in the median of the paired differences (change from baseline) was evaluated using the Wilcoxon matched pairs test. Percentage weight change was calculated by subtracting the weight at the final visit from the weight of the baseline visit, dividing by the weight of the baseline visit and multiplying by 100%. Summary responses to the app SUS (Figure 3) and DBWS SUS (Figure 4) are reported for study completers (in both calibration and intervention cohorts) as proportions of responses within each Likert category for each item in the questionnaire. The total score for these usability scales were calculated as described above and reported as medians with IQR.

**Table 1:** Baseline characteristics of study participants. Data reported for means (standard deviation) or median (interquartile range [IQR]).

Characteristics		Enrolled (N=55)	Calibration cohort (N=9)	Intervention cohort (N=38)
**Gender, n (%)**				
	Male	22 (40)	4 (44)	14 (37)
	Female	33 (60)	5 (56)	24 (63)
Age (years), mean (SD)		55.0 (10.6)	57.6 (0.9)	57.2 (−9.1)
Weight, kg, mean (SD)		92.47 (17.7)	92.1 (22.9)	92.6 (17.2)
**BMI^a^ (kg/m^2^), n (%)**				
	25-30	5 (9)	2 (22)	2 (5)
	30-35	14 (26)	3 (33)	10 (26)
	35-40	36 (65)	4 (45)	26 (69)
Waist circumference, cm, median (IQR)		106.7 (97.8-15.0)	100.3 (87.6-104.1)	108.2 (100.0-116.8)
A_1c_, %, median (IQR)		6.0 (5.7-6.2)	5.7 (5.7-6.0)	6.0 (5.7-6.2)
Fasting glucose, mg/dL, median (IQR)		102 (97-109)	98 (94-109)	105 (98-111)
**Race, n (%)**				
	Black	14 (25)	5 (56)	7 (18)
	White	39 (71)	4 (44)	31 (82)
	Other	2 (4)	0 (0)	0 (0)
**Education attainment, n (%)**				
	Less than high school	2 (4)	0 (0)	2 (5)
	High school graduate	8 (14)	0 (0)	7 (18)
	Some college	13 (24)	1 (11)	10 (26)
	Associates degree	1 (2)	0 (0)	1 (3)
	Bachelor’s degree	19 (34)	7 (78)	9 (24)
	Advanced degree	12 (22)	1 (11)	9 (24)
**Employment status, n (%)**				
	Part time (<40 hours)	14 (26)	2 (22)	10 (26)
	Full time (>40 hours)	27 (49)	3 (33)	21 (55)
	Unemployed	3 (5)	3 (33)	1 (3)
	Retired	10 (18)	2 (22)	6 (15)
	Disabled	1 (2)	1 (11)	0 (0)
**Comorbidities, n (%)**				
	Hypertension	26 (47)	4 (44)	19 (50)
	Hyperlipidemia	18 (33)	4 (44)	2 (5)
	Hypertriglyceridemia	2 (4)	0 (0)	12 (32)
Mobile phone usage attitudes score^b^, median (IQR)		88 (80-100)	88 (80-100)	87 (80-100)
**Physical activity stage of change, n (%)**				
	Precontemplation	0 (0)	0 (0)	0 (0)
	Contemplation	30 (54)	7 (78)	19 (50)
	Preparation	8 (15)	1 (11)	7 (20)
	Decision or action	8 (15)	1 (11)	5 (10)
	Maintenance	9 (16)	0 (0)	7 (20)

^a^BMI: body mass index.

^b^Survey questions in Multimedia Appendix 1.

**Table 2:** Secondary outcomes in intervention cohort (N=38). Data reported are means (standard deviation) or median (interquartile range [IQR]).

Outcome	Baseline	Final visit	Change from baseline	*P* value^a^
Physical activity, MET^b^-hours/week^c^, mean (SD)	14.6 (6.2)	17.4 (8.1)	2.8 (6.8)	.02
Weight, kg, mean (SD)	90.3 (17.2)	88.7 (17.2)	−1.6 (2.5)	<.001
Percentage weight change, mean (SD)	—	—	−1.9 (2.8)	—
BMI^d^, kg/m^2^, mean (SD)	32.6 (4.5)	31.9 (4.6)	−0.6 (0.8)	<.001
Waist circumference, cm, mean (SD)	109.0 (12.0)	107.6 (12.0)	−1.4 (2.9)	<.01
A_1c_, %, median (IQR)	6.00 (5.70-6.20)	5.85 (5.70-6.10)	−0.10 (−0.20 to 0.10)	.04
Fasting glucose, mg/dL, median (IQR)	106 (98-111)	102 (97-109)	−1 (−3 to 0)	.59
Systolic blood pressure, mm Hg, median (IQR)	131 (119-143)	129 (118-139)	1 (−10 to 7)	.56
Diastolic blood pressure, mm Hg, median (IQR)	77.5 (71-84)	75 (67-83)	−4 (−7 to 4)	.21

^a^Evaluated using paired *t* test for physical activity, weight, BMI, and waist circumference and Wilcoxon matched pairs (signed-rank) for A_1c_, fasting glucose, systolic blood pressure, and diastolic blood pressure.

^b^MET: metabolic equivalent of task.

^c^One observation missing from physical activity data because of inaccurate height entry into app by participant, resulting in inaccurate calculations of METs.

^d^BMI: body mass index.

## Results

### Study Population

Of participants who met the inclusion criteria by prescreening, 92 were assessed for eligibility by laboratory testing (Figure 2). After excluding 35 participants who had either normal glucose tolerance or diabetes mellitus, 57 participants were eligible for the study, of whom 55 (96%) enrolled. Of the 55 enrolled participants, 12 (22%) and 43 (78%) were in the calibration and intervention cohorts, respectively. Of the 12 participants in the calibration cohort, 9 (75%) completed the study according to protocol. One participant was dropped because he was logged out of the app for a period longer than 14 days, and 2 were dropped because of protocol violations (one participant failed to return for final visit, and another’s final visit was outside the study window). In the intervention cohort, 38 out of the 43 (88%) participants completed the study. Reasons for dropping out in this cohort included 3 participants that logged out or removed the app, one who voluntarily dropped out, and one protocol violation (torn knee meniscus that limited ambulation; notably, this participant did not attribute the knee injury to increased PA). Among the 55 enrolled participants, 14 (25%) received the Sweetch app alone, and 41 (75%) received the app + DBWS. Among the 47 participants who completed the study per protocol and were included in the analysis, 4 of 9 (44%) in the calibration cohort received both the app + DBWS and 31 of 38 (82%) in the intervention cohort received both the app + DBWS. Thus, 35 of the 47 (74%) study completers received both the app + DBWS.

The baseline characteristics of the study participants are shown in Table 1, summarized for all enrolled participants and separately for the calibration and intervention cohorts. Overall, participants were predominately female (60%), white (71%), middle aged (mean age 55.0 years [SD 10.6]), and obese (91% had BMI ≥30 kg/m^2^) adults with prediabetes. The median (IQR) baseline A_1c_ was 6.0% (5.7%-6.2%), and fasting glucose was 102 mg/dL (97-109). The diagnosis of prediabetes was established on the basis of impaired fasting glucose alone for 13/55 (24%), elevated A_1c_ alone for 21/55 (38%), and both criteria for 21/55 (38%) of enrolled participants. There was a high prevalence of cardiovascular risk factors overall, with 47% of participants having hypertension and 33% having hyperlipidemia. The majority (82%) of participants had at least some college education, and 75% were employed at least part time. The median score on the mobile phone usage and attitudes baseline survey was 88%, indicating that the participants had a high level of proclivity with basic mobile phone technology. Regarding PA stage of change, the majority of participants were in the contemplative stage (54%), defined as intending to become physically active in the next 6 months [21].

### Feasibility and Acceptability

With respect to feasibility measures, there was relatively high retention in the study, with 47 of 55 (86%) participants completing the study according to protocol. The median (IQR) time between the baseline and follow-up visits for study completers was 91 (90-98) days. Only one participant voluntarily withdrew from the study out of lack of interest. Despite referral to a dual-certified RD and CDE at the baseline visit, only 2 of 55 (4%) participants actually attended a visit for lifestyle counseling.

The Sweetch mobile platform had a high degree of acceptability by the participants. The median (IQR) score for the Sweetch app SUS measure was 78% (73%-80%), with a score above 68% indicating above average acceptability. The summary responses to the raw items used to calculate the app SUS measure are shown in Figure 3. The majority of participants agreed or strongly agreed that they would like to use the Sweetch app frequently (74%), found the app easy to use (83%), found the functions of the app well integrated (72%), felt that most people could learn to use the app very quickly (89%), and felt confident using the app (77%). Similarly, a majority of participants strongly disagreed or disagreed that the app was unnecessarily complex (81%), would need the support of a technical person (85%), had too much inconsistency (60%), found the app cumbersome to use (79%), and that they needed to learn a lot of things before being able to use the app (83%).

There was also high satisfaction with the DBWS with a median (IQR) usability score of 93% (83%-100%). The summary responses for the DBWS SUS measure are shown in Figure 4. The majority of participants strongly agreed or agreed that the DBWS was easy to use (94%) and connect to the mobile phone using Bluetooth (94%), found the weight measurements to be accurate (80%), did not have any technical challenges with the DBWS (85%), weight measures consistently synchronized to the mobile phone automatically (88%), and having automatic weight measurements on the mobile phone was helpful to achieving goals (77%). Comparing the perceived relative value of the app and DBWS in achieving their PA goal, 48% of participants found the app and DBWS equally effective, 46% found the app more effective than the DBWS, and 6% found the DBWS more helpful than the app. As a tool for weight loss, 72% found the app and DBWS equally effective, 14% found the app more helpful than then DBWS, 11% found the DBWS more helpful than the app, and 3% found neither to be helpful.

### Effectiveness and Safety

With respect to the effectiveness measures (Table 2), there was a significant increase in PA from baseline with a mean change of 2.8 MET-hours per week (SD 6.8; *P*=.02). Weight reduction of 1.6 kg (SD 2.5; *P*<.001) was observed, corresponding to weight change of approximately 2%. BMI declined by 0.6 kg/m^2^ (SD 0.8; *P*<.001), and waist circumference was reduced by 1.4 cm (SD 2.9; *P*<.01). There was a statistically significant and clinically meaningful reduction in A_1c_ in the intervention cohort, with median (IQR) A_1c_ change from baseline of −0.1% (−0.2% to 0.1%; *P*=.04). There was no significant change in the fasting glucose from baseline, with median (IQR) change of −1 mg/dL (−3 to 0; *P*=.59). There was no significant change in blood pressure with median (IQR) change of 1 mm Hg (−10 to −7; *P*=.56) and −4 mm Hg (−7 to 4; *P*=.21) in systolic and diastolic blood pressure, respectively. There were no AEs reported in this study. One participant had a knee injury (torn meniscus) approximately 30 days into the study, but this was unrelated to the Sweetch intervention.

## Discussion

### Principal Findings

This pilot study demonstrated that the Sweetch mobile platform in combination with a DBWS was effective at increasing PA and reducing weight and A_1c_ and had a high degree of acceptability. From a feasibility standpoint, there was a high rate of participant retention. Usability and satisfaction scores for the app and DBWS were uniformly high. As expected, given the nature of this intervention, there were no AEs.

The findings of this study suggest a potential role of fully automated mobile-based interventions for diabetes prevention. Participants achieved significant increases in PA, with a 2.8 MET-hour increase per week per participant. By comparison, the change in PA achieved at 3 and 6 months in the DPP study, which is considered the goal standard for diabetes prevention programs, was extrapolated to be 1.7 and 3.6 MET-hours per week, respectively. Participants lost an average of 1.6 kg, corresponding to approximately 2% weight loss at 3 months. The DPP showed that for each 1 kg weight loss in the lifestyle arm, there was a 16% reduction in diabetes risk, adjusted for changes in diet and activity [17]. The degree of weight loss achieved in this study was slightly less than the weight loss trend at the 3-month time point in the DPP; however, to sustain behavioral change, the Sweetch intervention sought to gradually expose participants to different diabetes prevention goals, starting with PA. As participants complied with the PA goal, a weight goal was subsequently introduced. Given the study interval of 3 months, the weight loss goal was introduced to participants later in the trial; hence, the smaller reduction in weight compared with the DPP. In this study, weight loss corresponded to a BMI reduction of 0.6 kg/m^2^; a large prospective cohort study showed that BMI reductions of at least 0.5 kg/m^2^ are associated with 12% reduction in the relative risk of incident diabetes over the long term [26].

With respect to glycemic measures, participants in the intervention cohort achieved A_1c_ reductions of 0.1% (*P*=.04), with no significant change in fasting glucose. The discordance between these glycemic measures may be explained by the fact that (1) A_1c_ is a more stable estimate of glycemic exposure than fasting glucose, (2) an intervention that increases PA may disproportionately improve postprandial hyperglycemia over fasting hyperglycemia because of improvements in muscle insulin sensitivity, and (3) unlike fasting glucose, A_1c_ results are not influenced by recent carbohydrate exposure and/or participant adherence to an 8-hour fast. Inferring from the results of the lifestyle arm in the DPP, an A_1c_ reduction of 0.1% is considered a clinically meaningful surrogate for diabetes risk: at 3 and 6 months, the change in A_1c_ from baseline in the DPP were −0.05% and −0.1%, respectively [10]. Thus, the A_1c_ change realized at the 3-month time point in this study exceeded the change at 3 months that was extrapolated from the lifestyle arm of the DPP. It is important to note that in this study, all participants were referred for lifestyle education with a CDE and RD, but only 2 of 55 (4%) participants actually attended a visit for lifestyle counseling; therefore, it is likely that the findings can be attributed almost entirely to the Sweetch intervention. Although there was no significant change in blood pressure, this was not the main objective of this study.

### Significance of Findings

This study adds to a growing body of evidence supporting the use of mHealth technology for improving PA levels, weight loss, and diabetes prevention [12]. A mobile phone or Web-based DPP (Alive-PD) that included tailored goal setting, weekly tracking, human-based phone coaching, and twice-weekly DPP curriculum achieved reductions of 0.26% and 3.3 kg weight loss at 6 months [27]. Another intervention that included in-person DPP curriculum sessions and a mobile app for self-monitoring of weight, activity, and diet resulted in 6.2 kg weight loss at 5 months but failed to demonstrate any significant change in glycemic measures [14]. A hybrid program combining human coaching and digital tools, Noom Coach (Noom, Inc, New York), demonstrated significant weight loss but did not report glycemic data [15]. Nonetheless, given the significant effect on weight reduction seen with this intervention, the CDC included Noom in their recently established DPP recognition registry [28]. Omada Health (San Francisco), another solution that combines human coaching and digital tools, also demonstrated significant weight loss, but no change in A_1c_ was observed at 3 months [29]. On the other hand, the intervention demonstrated significant A_1c_ reductions of 0.37% at 12 months (*P*=.001).

The fact that our study demonstrated both weight and A_1c_ reductions at only 3 months suggests that long-term effects will be comparable, if not superior, to existing interventions. Most importantly, Sweetch’s machine learning technology enables fully automated intervention; hence, supporting larger-scale deployment with greater cost-effectiveness potential when compared with human-based diabetes prevention solutions. A future long-term study with the Sweetch mobile platform is planned to confirm the durability of these findings and to satisfy CDC recognition criteria for a DPP.

### Study Strengths and Limitations

There were several strengths to this study. PA measures were tracked, rather than self-reported, which increases the validity of this measure as compared with the DPP that relied on participant self-report of PA. Our study used the DPP study as a comparison, as this study is considered the gold standard for diabetes prevention. Unlike a similar study that used home A_1c_ test kits and thus received A_1c_ data on only 53% of participants at study follow-up [30], we had complete ascertainment of A_1c_ levels on study completers. That said, one of the lessons learned from this study is that point-of-care A_1c_ screening would be more efficient for recruitment and would enhance the implementation of this intervention in a real-world clinical setting where patients identified as having prediabetes could receive the intervention immediately without the need for fasting and a return visit. With respect to the app itself, another lesson learned from this study is that adding a component focused on nutrition in addition to weight loss and PA goals may be required to achieve maximal weight loss and greater improvements in glycemic measures.

Limitations of this study were the single-arm design with a self-selected sample of participants who may be more motivated to change their lifestyle than the average patient with prediabetes. Although the focus of this intervention was weight loss and PA, participants did receive nutrition information (brochure) at the baseline visit, which may have affected the intervention. Our patient demographic consisted predominately of white females, which is not uncommon in behavioral weight loss studies. Although the study enrolled overweight and obese adults, the study population consisted of predominantly obese individuals who may have led a more sedentary lifestyle than their overweight counterparts. A further study with a larger sample size will allow inferences to be made about the generalizability of these findings to overweight prediabetic patients. Finally, although the aim of the app is diabetes prevention, given the size and duration of this study, we were limited to using surrogate measures (ie, PA, weight change, and A_1c_) to draw inferences about diabetes risk, rather than measuring diabetes incidence.

### Conclusions

The Sweetch mobile platform was well received by participants and was effective at increasing PA and reducing body weight and A_1c_ over 3 months without any adverse effects. The study results are promising, but future studies will be required to confirm the sustainability of these findings over a longer follow-up period.

## Appendix

Multimedia Appendix 1 Mobile phone usage and attitudes baseline survey.

Multimedia Appendix 2 Calculation of MET-hours per week per participant.

## Abbreviations

AE: adverse event
BMI: body mass index
CDC: Centers for Disease Control and Prevention
CDE: certified diabetes educator
DBWS: digital body weight scale
DPP: Diabetes Prevention Program
IQR: interquartile range
IRB: institutional review board
LTPA: leisure time physical activity
MET: metabolic equivalent of task
mHealth: mobile health
PA: physical activity
RCT: randomized controlled trial
RD: registered dietician
SUS: System Usability Scale
