# Supplementary material for: A Novel Approach for Fully Automated, Personalized Health Coaching for Adults with Prediabetes: Pilot Clinical Trial
Source: J Med Internet Res. 2018 Feb 27;20(2):e72. doi: 10.2196/jmir.9723 (PMC5849796; doi:10.2196/jmir.9723)
Supplement: Multimedia Appendix 1 [file jmir_v20i2e72_app1.pdf]

Supplemental Table 1. Mobile Phone Usage and Attitudes Baseline Survey

Please indicate how often you do each of the following activities on your mobile phone.

1. Send and receive text messages.

Never (1) Once a month (2) Once a week (3) Once a day (4) All the time (5)

2. Make and receive mobile phone calls.

Never (1) Once a month (2) Once a week (3) Once a day (4) All the time (5)

3. Get directions or use GPS.

Never (1) Once a month (2) Once a week (3) Once a day (4) All the time (5)

4. Browse the web.

Never (1) Once a month (2) Once a week (3) Once a day (4) All the time (5)

5. Use apps (for any purpose) on mobile phone.

Never (1) Once a month (2) Once a week (3) Once a day (4) All the time (5)

Scoring:

Value of responses (in parentheses) is multiplied by 4 and total score equals sum of responses. Minimum score = 20; Maximum score=100.
