# Supplementary material for: A Novel Approach for Fully Automated, Personalized Health Coaching for Adults with Prediabetes: Pilot Clinical Trial
Source: J Med Internet Res. 2018 Feb 27;20(2):e72. doi: 10.2196/jmir.9723 (PMC5849796; doi:10.2196/jmir.9723)
Supplement: Multimedia Appendix 2 [file jmir_v20i2e72_app2.pdf]

Supplemental Table 2. Calculation of MET-hours per week per participant.

Step	Description	Equation	Example
1	Calculate stride length in cm	Height(cm) * 0.414[a]	173.5 * 0.414= 71.829 cm
2	Convert stride length to inches	Stride length (cm) * 1 in/2.5 cm	71.298/ 2.5= 28.73 in
3	Convert stride length to feet	Stride length (in) * 1 ft/ 12 inc	27.73/12= 2.39 ft
4	Calculate steps per mile	5,280 feet / stride length	5,280/ 2.39= 2205 steps
5	Convert to miles per step	1 / (steps/mile)	1/ 2205= 0.0004535 miles/step
6	Calculate steps per min of activity[b]	Steps/ duration of activity (min)	108 steps/ 2 min= 54 steps/min
7	Convert steps per minute to steps per hour	Steps per min * 60 min/hour	54 * 60= 3,240 steps/hour
8	Calculate miles per hour (speed)	Steps per hour * miles / step	3,240 * 0.0004535 = 1.5 mph
9	Convert to METs based on activity type[c] and speed (mph) from step 8	MET Values[2]:<br>Walking:<br><2.0 mph: 2 METs<br>2.0 to <2.5 mph:2.8 METs<br>2.5 to <2.8 mph: 3.0 METs<br>2.8 to <3.2 mph: 3.5 METs<br>3.2 to <3.5 mph: 4.3 METs<br>3.5 to <4.0 mph: 5.0 METs<br>4.0 to <4.5 mph: 7.0 METs<br>4.5 to <5.0 mph: 8.3 METs<br><br>Running:<br><4.0 mph: 6.0 METs<br>4.0 to <5.0 mph: 8.3 METs<br>5.0 to <5.2 mph: 9.0 METs<br>5.2 to <6.0 mph: 9.8 METs<br>6.0 to <6.7 mph: 10.5 METs<br>6.7 to <7.0 mph: 11.0 METs<br>7.0 to 8.0 mph: 11.8 METs<br>8.0 to <8.6 mph: 12.3 METs<br>8.6 to <9.0 mph: 12.8 METs<br>9.0 to <10.0 mph: 14.5 METs<br>10.0 to <11.0 mph: 16.0 METs<br><br>Cycling:<br>5.8 METs (average cycling speed)	Activity Type= Walking<br>1.5 mph = 2 METs per conversion table
10	Calculate Met*min	MET (step 9) * activity duration (min)	2 METs * 2 min= 4 MET*min
11	Convert to Met*hr	MET*min * 1 hour/60 min	4 /60= 0.067 met/hr
12	Sum Met*hrs of all activity		

	segments per week per participant		

[a] Average stride length for females is height X 0.413 and for males is height x 0.415 For simplicity, the average conversion (0.414) was used for all participants to estimate stride length[1]
[b] Steps and activity segment duration captured on smartphone using built in pedometer/accelerometer from smartphone
[c] Activity type categorized as walking, running, or cycling and determined by sponsor based on pedometer/accelerometer data from smartphone

Supplemental Table References:

1. Grieve DW, Gear RJ. The relationships between length of stride, step frequency, time of swing, and speed of walking for children and adults. Ergonomics. 1966 Sep:9(5);379-99.
2. MET values derived from the published online values from the updated 2011 Adult Compendium of Physical Activities, supported by Arizona State University and the National Cancer Institute. https://sites.google.com/site/compendiumofphysicalactivities/home. Accessed December 8, 2017.
